# Direct Observation of Inner-Layer Inward Contractions of Multiwalled Boron Nitride Nanotubes upon in Situ Heating

**DOI:** 10.3390/nano8020086

**Published:** 2018-02-04

**Authors:** Zhongwen Li, Zi-An Li, Shuaishuai Sun, Dingguo Zheng, Hong Wang, Huanfang Tian, Huaixin Yang, Xuedong Bai, Jianqi Li

**Affiliations:** 1 Beijing National Laboratory for Condensed Matter Physics, Institute of Physics, Chinese Academy of Sciences, Beijing 100190, China; lizhongwen@iphy.ac.cn (Z.L.); sss@iphy.ac.cn (S.S.); zdg@iphy.ac.cn (D.Z.); hong.w@iphy.ac.cn (H.W.); hftian@iphy.ac.cn (H.T.); hxyang@iphy.ac.cn (H.Y.); xdbai@iphy.ac.cn (X.B.); 2 School of Physical Sciences, University of Chinese Academy of Sciences, Beijing 100049, China; 3 Collaborative Innovation Center of Quantum Matter, Beijing 100084, China

**Keywords:** multi-walled BNNTs, anisotropic thermal expansion, transmission electron microscopy, in situ heating, thermal contraction

## Abstract

In situ heating transmission electron microscopy observations clearly reveal remarkable interlayer expansion and inner-layer inward contraction in multi-walled boron nitride nanotubes (BNNTs) as the specimen temperature increases. We interpreted the observed inward contraction as being due to the presence of the strong constraints of the outer layers on radial expansion in the tubular structure upon in situ heating. The increase in specimen temperature upon heating can create pressure and stress toward the tubular center, which drive the lattice motion and yield inner diameter contraction for the multi-walled BNNTs. Using a simple model involving a wave-like pattern of layer-wise distortion, we discuss these peculiar structural alterations and the anisotropic thermal expansion properties of the tubular structures. Moreover, our in situ atomic images also reveal Russian-doll-type BN nanotubes, which show anisotropic thermal expansion behaviors.

## 1. Introduction

In the recent decades, one-dimensional (1D) tubular nanomaterials, notably the carbon-based nanotubes and the mineral-based nanotubes [1,2,3], have attracted a great deal of attention for many applications because of their novel physical properties [4,5,6]. Carbon nanotubes (CNTs) are one prominent example, and exhibit rich transport properties including insulating, semiconducting and metallic behaviors, depending on the tubular chirality [7]. Boron nitride nanotubes (BNNTs), structurally similar to CNTs, also have tubular structures, but only exhibit semiconducting transport with a large band gap, regardless of tubular chirality [8,9]. BNNTs also exhibit high chemical stability, excellent mechanical properties, and high thermal conductivity. These unique properties make BN nanotubes a promising nanomaterial in a variety of potential fields, such as gas sensing, spin filters, bio sensing, functional composites, hydrogen accumulators, and electrically insulating substrates [9,10,11,12,13]. Exploitation of nanoscale electronic devices based on these tubular nanostructures requires a full understanding of not only the static structural characteristics, but also the structural response to externally applied stimuli [14], e.g., thermal heat and electromagnetic waves. However, the nanosized tubular structure imposes great challenges in investigating their structural response on the individual nanotube level.

High-resolution transmission electron microscopy (HRTEM) allows a direct imaging of the atomic column of thin specimens, and has become an indispensable tool for studying nanosized materials at an atomic resolution. In recent years, in situ experimentation in HRTEM has emerged as an exciting field of study [15]. The application of HRTEM to studying dynamic changes in a specimen at atomic scale have met with challenges due to the lack of mechanical stability of specimens when applying stimuli, e.g., in situ heating. Recently, with the advancements in microelectromechanical systems (MEMS)-based technologies, in situ HRTEM studies have progressed rapidly over the last decade. Here, we employ the MEMS-based in situ heating holder to study the thermal properties of BNNTs, with a particular focus on the atomic-resolution layer-by-layer lattice change due to in situ heating. We aim to understand the peculiar thermal expansion in the interlayer of van der Waals bonds and the thermal contraction in the intralayer of strong covalent bonds in the tubular BNNTs.

## 2. Experimental

The in situ heating TEM observations were performed on a Jeol-2100F TEM (JEOL Ltd., Tokyo, Japan) operating under an acceleration voltage of 200 kV. A chip-type heating TEM holder (Protochip, Inc., Morrisville, NC, USA) was used to obtain the electron diffraction and high-resolution lattice images at high temperatures. Our samples of BNNTs were synthesized by Advanced Materials Laboratory, National Institute for Materials Science (NIMS), Japan. More information about the samples can be obtained from ref. [16,17]. The multi-walled BN nanotubes were dispersed in ethanol using an ultrasonicator for half an hour. Then the mixed liquid was dropped on the micro-grid of the heating holder. The local sample temperature can be varied from room temperature to 1200 K. For accurate lattice measurement with high precision, the TEM imaging and diffraction conditions and the specimen positions are kept nearly identical at different specimen temperatures.

## 3. Results and Discussion

**Figure 1:**
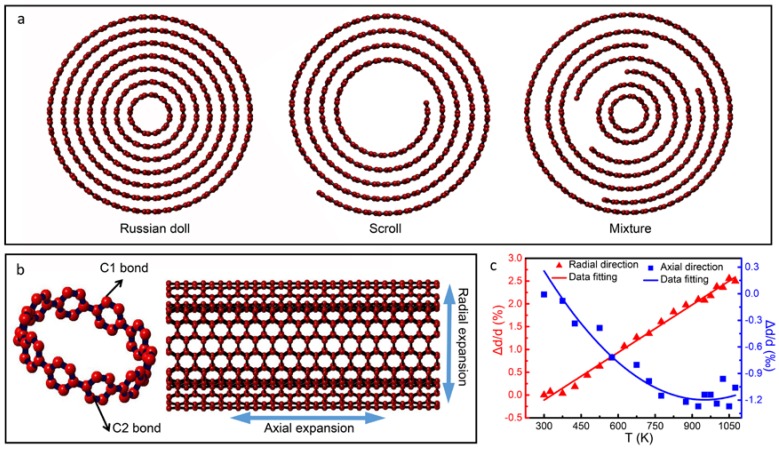
Atomic models for tubular structure, the radial and axial directions are indicated. (**a**) Three types of BN tubular structure: Russian doll, scroll and the mixture; (**b**) Lattice change in the radial and axial directions in nanotubes of Russian doll; (**c**) Thermal coefficient of multi-walled boron nitride nanotubes using the in situ TEM heating experiment.

Prior to discussing the experimental results, we will firstly discuss the three tubular atomic models of BN nanotube in the literature. It is commonly known that multi-walled nanotubes have three types of tubular structures, i.e., Russian doll, jelly scroll, and mixture structures [18], as shown in Figure 1a. They have all been directly observed in transmission electron microscope (TEM) investigations [19]. Measurements of thermal expansions by means of X-ray and electron scatterings have clearly demonstrated that the interlayer spacings between atomic sheets often increase evidently from (*l*) to (*l* + Δ*l*) upon the increase of temperature due to the anharmonic nature of inter-atomic Van der Waals interaction.

For the doll model, we can take the armchair nanotube as an example, which consists of C1 (circumferential) and C2 (axial) bonds [20], as shown in Figure 1b. If we only consider the variation of nanotubes caused by the alterations of chemical bonds during temperature rise, the variation of axial length should be comparable with that along the radial direction, which can be estimated by (1 + Δ*l*/*l*) times the original length. For the Russian doll model, as shown in Figure 1b, the thermal expansion in the axial and radial directions should adopt a similar feature. For the Jelly scroll and the mixture models, anisotropic lattice expansions can be observed, and visibly large lattice expansion occurs in the radial direction due to the weak Van der Waals interaction between layers. On the other hand, based on a large number of results from XRD and TEM experimental observations, nanotubes often show notable anisotropic characteristics during thermal expansion. For example, the thermal expansion coefficient of carbon nanotubes in the radial direction is 2.5 × 10^−5^ K^−1^, while the lattice expansion in the axial direction is only 1/10 of the former [18,21]. Figure 1c shows the temperature-dependent lattice changes in the axial and radial directions obtained from a series of electron diffraction patterns from 300 to 1100 K by in situ heating of BNNTs inside the TEM. The BNNTs exhibit a notable linear expansion in the radial direction, and the coefficient of expansion is measured to be 3.5 × 10^−5^ K^−1^. By contrast, the axial direction exhibits a negative thermal expansion behavior, which is fitted with a two-term polynomial function [22]. These data are comparable with those obtained from graphite and H-BN crystals [23]. Therefore, the main experimental results, in particular the large inter-sheet thermo-expansion, are interpreted to be based on Jelly scroll and mixture models [18,24,25]. However, according to our recent study on the microstructures of a number of well-characterized samples, a large fraction of the BNNTs adopt the Russian doll structure. Moreover, these multi-walled nanotubes exhibit visible inner diameter contraction associated with lattice expansion as temperature increases.

**Figure 2:**
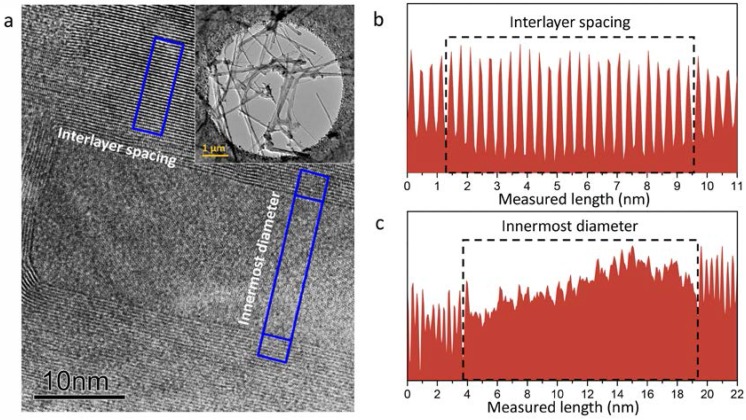
High-resolution lattice image of multi-walled boron nitride nanotubes BNNTs. (**a**) High-resolution lattice images of BNNTs, in which the interlayer spacing and the diameter of the innermost layer are indicated. The inset shows a low-magnification TEM image of the BNNT assembly; Line-scan profiles trace the interlayer spacing in (**b**), and the diameter of the innermost layer in (**c**).

Figure 2 shows the atomic-resolved lattice images obtained as a function of applied temperature, from which the interlayer spacing and the diameter of the innermost layer are measured [26]. Figure 2a shows a typical high-resolution image of BNNTs at room temperature, in which the lattice spacing, as well the innermost layer diameters for the measured tubular, can be simultaneously obtained. Figure 2b shows the line profile traces of the high-resolution lattice image in the direction perpendicular to the nanotube axis (boxed area). To improve the lattice measurement accuracy, the broad width of the scan line is used, as indicated by the boxed area. The periodical line profiles trace the tubular layers and interlayer spacing of the multi-walled BNNTs. Figure 2c illustrates the measurement of the diameter of the innermost layer of BNNTs, as measured directly between the two innermost layers. We note that in situ heating of the specimen with varying temperature leads to a slight specimen drifts. The high-resolution images were taken after the pre-set temperature was reached and the specimen was found to be stabilized. We use the specific structural features in the nanotube as a reference mark, and the lattice images of different temperature were realigned prior to lattice measurement. In order to reduce the degree of experimental error, we used average data taken from a series of atomic-resolved images at the same temperature for the data processing.

**Figure 3:**
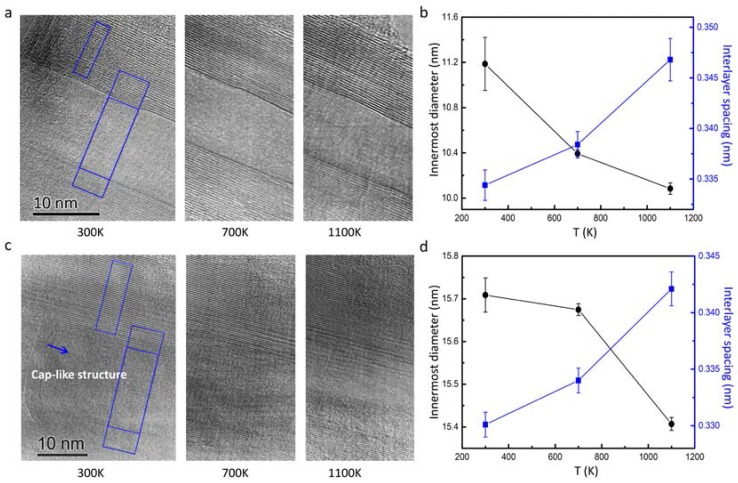
High-resolution lattice images of BNNTs from in situ TEM heating experiments. (**a**) High-resolution lattice images of BNNTs taken at 300, 700 and 1100 K, respectively; (**b**) the corresponding lattice spacings of the axial and radial directions for the three temperatures; (**c**,**d**) are the same as (**a**,**b**), but for nanotubes with a cap-like structures.

Three sets of in situ heating experimental results from room temperature up to 1100 K are shown in Figure 3. Figure 3a shows in situ heating experimental images for a multi-walled BNNT. Using the line-scan profile method as described in Figure 2, we measured the interlayer spacing and the diameter of the innermost layer from Figure 3a, as shown in Figure 3b. It is recognizable that the innermost diameter decreases progressively with the temperature rise. In contrast, the interlayer spacings become larger with temperature rise. Importantly, the experimental results for layer spacing modification are basically consistent with the data calculated by using thermal expansion with the coefficient as discussed in the above context. This fact suggests that the in situ heating TEM images reliably reveal the lattice expansion in the multi-walled tubular structure. At a temperature of 1100 K, the experimental data for the inner diameter of this nanotube contracts by 1.1 nm (11%) in comparison with the room temperature data.

Figure 3c shows a second sample with an additional cap-like tubular structure. Figure 3d presents the interlayer spacing and innermost diameter as a function of temperature. It is notable that the second nanotube has a closed cap-like structure in the inner layer, which actually features the multi-walled BN nanotube for the Russian doll structure, as reported for BN tubes in the microstructural analysis. The inner diameter decreases as temperature increases, and the lattice distance between interlayers increases as temperature increases. The changes of the interlayer spacing of this nanotube show a fundamentally similar feature with the previous one. However, the contraction of the innermost diameter (~3%) is much smaller than that (11%) of the first one (Figure 3a,b). Obviously, the cap-like tubular structure inside the tube of the second sample (see Figure 3c) play a role in hindering the inward contraction of the nanotube.

It is known that the interlayer spacing of multi-walled BN nanotubes depends mainly on the weak Van der Waals interaction, as extensively discussed in the previous literature. This can be greatly affected by the morphologic features of specific BN sheets in nano-tubular structures [27,28]. In the present case, we suggest that the strong constraints exerted by the outer layers prevent the lattice expansion of multi-walled BNNTs in the radially outward direction. Conversely, the temperature rise could create pressure and stress on the inner layers of the tubes, and this type of stress could release progressively with the BN inner-layer motions radially inward to the tubular center, i.e., the contraction of the innermost layers associated with the increase in temperature, as seen in our in situ heating TEM observations. In fact, similar experimental phenomena have been noted in metal-filled multi-walled carbon nanotubes. Sun and coworkers have reported that, with increasing specimen temperature, the metal or carbide nanowires inside the carbon nanotube are subjected to an inner pressure of up to 40 GPa because of the contraction of inner tubular layers [29].

Admittedly, using static high-resolution atomically resolved imaging in the present study, it is still difficult to obtain a microscopic understanding of the structural distortion and lattice motions occurring in the inner tubular layers following in situ heating. The contraction in axial direction of the BN nanotubes (Figure 1c) is generally believed to be caused by certain anharmonic phonon modes of the multi-walled nanotubes [30]. Therefore, we turn to a descriptive model discussion based on the morphology alterations associated with thermo-excitation of phonon modes in tubular structure [31]. The phonon modes of nanotubes can be categorized into three types: torsional, longitudinal and radial modes. Because the interlayer Van der Waals interaction is determined by the radial direction only, it has almost no effect on torsional and longitudinal mode [32,33].

**Figure 4:**
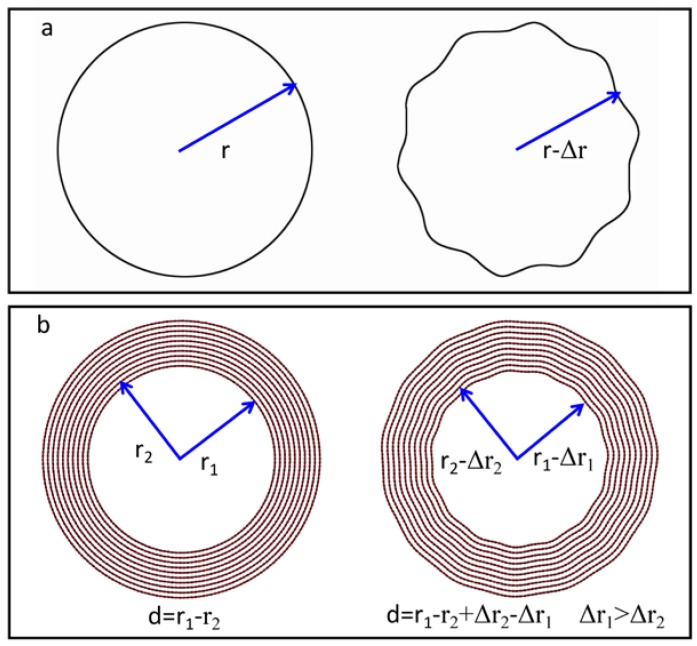
Schematic models for the contraction of innermost layer of nanotube due to the heating effect on the tubular structure (in the cross-section view). (**a**) The left-panel shows the schematic diagram without the wave-like atomic displacement, and the right-panel shows the lattice contraction caused by the wave-like atomic displacement; (**b**) Illustrative schematics of lattice contraction of innermost layers of multi-walled tubular structure upon in situ heating.

The radial modes have major effects, and it is shown that the radial mode can cause the contraction of the inner diameter of the nanotubes, whereby the atoms on the cross-section of the nanotubes can be defined as follows (Figure 4a),

(1) $$r_{1}=r(T)+A\cdot \sin(\omega \cdot \theta )$$

where *r*_1_ is the distance from each atom to the center of the cross-section, *r*(*T*) is the average distance from each atom to the center at a certain temperature *T*, and *A* is the amplitude of displacement, and 1/*ω* is wavelength of the phonon mode. Obviously, the inner diameter of the nanotube exhibits clear contraction in comparison with the outer layers. We then do the following calculations,

(2) $$2\pi r_{0}=\int_{0}^{2\pi }\sqrt{dr_{1}^{2}+{\left(r_{1}\cdot d\theta \right)}^{2}}$$

where *r*_0_ is the radius of the nanotube at 0 K. In our experiments, *r_1_* is much larger than *A*, so the following results can be obtained when Equation (2) is expanded by the Taylor formula, neglecting the higher-order term.

(3) $$r_{0}-r(T)=\frac{(2\omega^{2}-1)A^{2}}{r_{0}}$$

where *r*_0_ − *r*(*T*) is the contraction, Δ*r*, of the nanotube relative to 0 K. As a result, the smaller the inner diameter of the monolayer multi-wall nanotubes is, the greater the shrinkage that occurs, as shown in Figure 4b. Generally speaking, with a rise in temperature, there is no new vibration mode or frequency activated; instead, only the vibration amplitude increases [34]. The interlayer spacing of the nanotubes will become larger due to the shrinkage of the inner layer driven by increasing specimen temperature.

The *A*^2^ can be considered to be linearly increasing upon temperature rise. When the specimen temperature increases, the inner-layer diameter of the tubular structure tends to shrink, and the interlayer spacing will increase accordingly. We therefore suggest that the structural evolutions of the multi-walled BN nanotubes evidently depend on both the inter-layer bonds and the strong restriction arising initially from the outer tubular layers, which explains well the anisotropic thermal expansion of the nanotubes observed in our in situ heating experiment.

## 4. Conclusions

Using in situ high-resolution TEM and the MEMS-based heating holder, we have studied the thermal lattice change in the multi-walled boron nitride nanotubes (BNNTs). Analysis of the atomically resolved lattice images at different applied temperature reveals the inner-layer contraction of the BN nanotube with an increase in specimen temperature. To understand the inner-layer inward contraction, we performed a simple model calculation to illustrate the structural alterations associated with the thermal expansion in multi-walled boron nitride tubes of doll model. Based on the model calculation, we interpret the observed inward contraction as being due to the presence of strong constraints of the outer-layers on the radial expansion in the tubular structure upon in situ heating.
